# 6-(4-Bromo­phen­yl)-3-methyl-7*H*-1,2,4-triazolo[3,4-*b*][1,3,4]thia­diazine

**DOI:** 10.1107/S1600536812019885

**Published:** 2012-05-12

**Authors:** Hoong-Kun Fun, Ching Kheng Quah, Hatem A. Abdel-Aziz, Mohamed I. Attia

**Affiliations:** a X-ray Crystallography Unit, School of Physics, Universiti Sains Malaysia, 11800 USM, Penang, Malaysia; b Department of Pharmaceutical Chemistry, College of Pharmacy, King Saud University, PO Box 2457, Riyadh 11451, Saudi Arabia

## Abstract

In the title compound, C_11_H_9_BrN_4_S, the 1,2,4-triazole ring is essentially planar (r.m.s. deviation = 0.020 Å) and makes a dihedral angle of 29.1 (5)° with the bromo­benzene ring. The 3,6-dihydro-1,3,4-thia­diazine ring adopts a twist-boat conformation. In the crystal, mol­ecules are linked by C—H⋯N inter­actions into sheets lying parallel to the (010) plane. The same N atom accepts two such hydrogen bonds.

## Related literature

For general background to and the chemistry and biological activity of the title compound, see: Holla *et al.* (2001 ▶); Prasad *et al.* (1998 ▶); Dawood *et al.* (2005 ▶); Abdel-Aziz *et al.* (2007 ▶); Abdel-Wahab *et al.* (2009 ▶). For further synthesis details, see: Dickinson & Jacobsen (1975 ▶). For standard bond-length data, see: Allen *et al.* (1987 ▶). For the stability of the temperature controller used in the data collection, see: Cosier & Glazer (1986 ▶). For ring conformations, see: Cremer & Pople (1975 ▶).
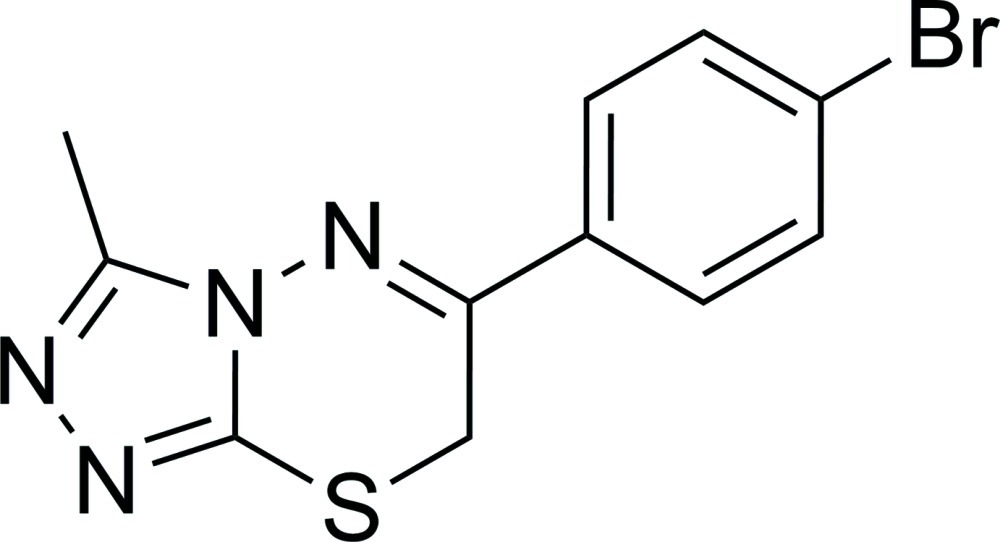


## Experimental

### 

#### Crystal data

C_11_H_9_BrN_4_S
*M* *_r_* = 309.19
Monoclinic, 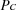

*a* = 4.0047 (10) Å
*b* = 13.424 (3) Å
*c* = 10.938 (3) Å
β = 99.650 (5)°
*V* = 579.7 (2) Å^3^
*Z* = 2
Mo *K*α radiation
μ = 3.71 mm^−1^
*T* = 100 K
0.46 × 0.10 × 0.03 mm

#### Data collection

Bruker SMART APEXII CCD diffractometer
Absorption correction: multi-scan (*SADABS*; Bruker, 2009 ▶) *T*_min_ = 0.281, *T*_max_ = 0.910
5127 measured reflections
2087 independent reflections
1904 reflections with *I* > 2σ(*I*)
*R*_int_ = 0.048

#### Refinement

*R*[*F* ^2^ > 2σ(*F* ^2^)] = 0.057
*wR*(*F* ^2^) = 0.152
*S* = 1.04
2087 reflections
149 parameters
2 restraints
H-atom parameters constrained
Δρ_max_ = 3.33 e Å^−3^
Δρ_min_ = −0.86 e Å^−3^
Absolute structure: Flack (1983 ▶), 950 Friedel pairs
Flack parameter: 0.01 (2)

### 

Data collection: *APEX2* (Bruker, 2009 ▶); cell refinement: *SAINT* (Bruker, 2009 ▶); data reduction: *SAINT*; program(s) used to solve structure: *SHELXTL* (Sheldrick, 2008 ▶); program(s) used to refine structure: *SHELXTL*; molecular graphics: *SHELXTL*; software used to prepare material for publication: *SHELXTL* and *PLATON* (Spek, 2009 ▶).

## Supplementary Material

Crystal structure: contains datablock(s) global, I. DOI: 10.1107/S1600536812019885/hb6774sup1.cif

Structure factors: contains datablock(s) I. DOI: 10.1107/S1600536812019885/hb6774Isup2.hkl

Supplementary material file. DOI: 10.1107/S1600536812019885/hb6774Isup3.cml

Additional supplementary materials:  crystallographic information; 3D view; checkCIF report

## Figures and Tables

**Table 1:** Hydrogen-bond geometry (Å, °)

*D*—H⋯*A*	*D*—H	H⋯*A*	*D*⋯*A*	*D*—H⋯*A*
C8—H8*A*⋯N3^i^	0.97	2.56	3.185 (12)	122
C8—H8*B*⋯N3^ii^	0.97	2.31	3.191 (12)	151

Symmetry codes: (i) 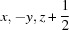; (ii) 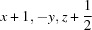.

**Fig. 1.**
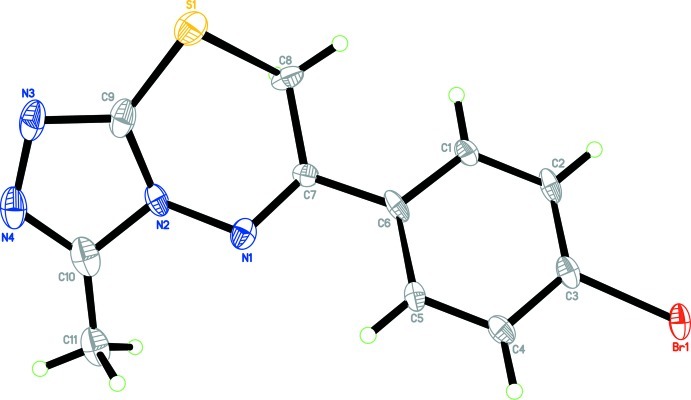
The molecular structure of the title compound showing 50% probability displacement ellipsoids for non-H atoms.

**Fig. 2.**
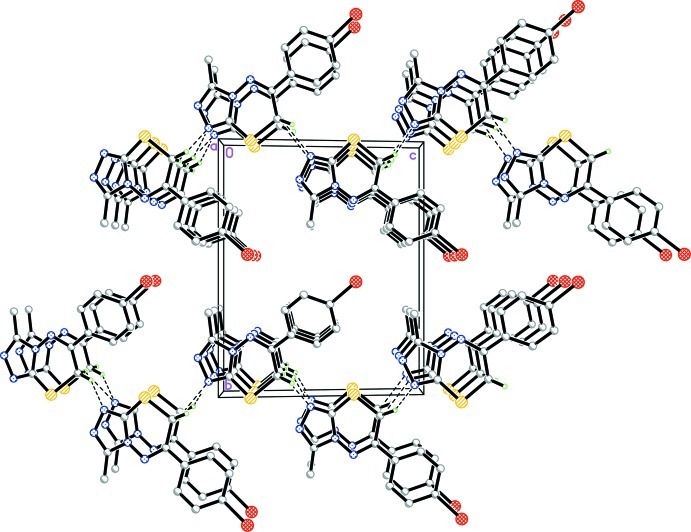
The crystal structure of the title compound, viewed along the a axis. H atoms not involved in hydrogen bonds (dashed lines) have been omitted for clarity.

## supplementary crystallographic information

### Comment

For the background of the chemistry of 1,2,4-triazolo[3,4-*b*]-1,3,4-thiadiazine derivatives see: Holla *et al.* (2001) & Prasad *et al.* (1998). In continuation of our studies in the chemistry and biological activities of the title compound analogs (Dawood *et al.*, 2005; Abdel-Aziz *et al.*, 2007 & Abdel-Wahab *et al.*, 2009), we reported the synthesis and crystal structure of the title compound.

In the title compound, Fig. 1, the 1,2,4-triazole (N2-N4/C9/C10) is essentially planar (r.m.s. deviation = 0.020 Å) and makes a dihedral angle of 29.1 (5)° with the phenyl ring (C1-C6). The 3,6-dihydro-1,3,4-thiadiazine ring (S1/N1/N2/C7-C9) adopts a twist-boat conformation, with puckering parameters Q = 0.552 (8) Å, Θ = 66.6 (9)° and φ = 32.5 (10)° (Cremer & Pople, 1975). Bond lengths (Allen *et al.*, 1987) and angles are within normal ranges.

In the crystal (Fig.2), molecules are linked *via* C8–H8A···N3 and C8–H8B···N3 bonds (Table 1) into sheets parallel to the (010) plane.

### Experimental

The reaction of 4-amino-5-methyl-4*H*-1,2,4-triazole-3-thiol and 2-bromo-1-phenylethanone afforded the title compound in the form of colourless plates according to the reported method (Dickinson & Jacobsen, 1975).

### Refinement

All H atoms were positioned geometrically and refined using a riding model with C–H = 0.93-0.97 Å and *U*_iso_(H) = 1.2 or 1.5 *U*_eq_(C). A rotating-group model was applied for the methyl group. The same U_ij_ parameters were used for atom pair C10/C11. The highest difference peak is 0.97Å from Br1. The deepest difference hole is 0.89Å from Br1.

### Crystal data

**Table:**

C_11_H_9_BrN_4_S	*F*(000) = 308
*M**_r_* = 309.19	*D*_x_ = 1.771 Mg m^−^^3^
Monoclinic, *P**c*	Mo *K*α radiation, λ = 0.71073 Å
Hall symbol: P -2yc	Cell parameters from 2139 reflections
*a* = 4.0047 (10) Å	θ = 2.4–27.8°
*b* = 13.424 (3) Å	µ = 3.71 mm^−^^1^
*c* = 10.938 (3) Å	*T* = 100 K
β = 99.650 (5)°	Plate, colourless
*V* = 579.7 (2) Å^3^	0.46 × 0.10 × 0.03 mm
*Z* = 2	

### Data collection

**Table:**

Bruker SMART APEXII CCD diffractometer	2087 independent reflections
Radiation source: fine-focus sealed tube	1904 reflections with *I* > 2σ(*I*)
Graphite monochromator	*R*_int_ = 0.048
φ and ω scans	θ_max_ = 26.0°, θ_min_ = 1.5°
Absorption correction: multi-scan (*SADABS*; Bruker, 2009)	*h* = −4→4
*T*_min_ = 0.281, *T*_max_ = 0.910	*k* = −15→16
5127 measured reflections	*l* = −13→12

### Refinement

**Table:**

Refinement on *F*^2^	Secondary atom site location: difference Fourier map
Least-squares matrix: full	Hydrogen site location: inferred from neighbouring sites
*R*[*F*^2^ > 2σ(*F*^2^)] = 0.057	H-atom parameters constrained
*wR*(*F*^2^) = 0.152	*w* = 1/[σ^2^(*F*_o_^2^) + (0.1109*P*)^2^] where *P* = (*F*_o_^2^ + 2*F*_c_^2^)/3
*S* = 1.04	(Δ/σ)_max_ = 0.001
2087 reflections	Δρ_max_ = 3.33 e Å^−^^3^
149 parameters	Δρ_min_ = −0.86 e Å^−^^3^
2 restraints	Absolute structure: Flack (1983), 950 Friedel pairs
Primary atom site location: structure-invariant direct methods	Flack parameter: 0.01 (2)

### Special details

**Table:**

Experimental. The crystal was placed in the cold stream of an Oxford Cryosystems Cobra open-flow nitrogen cryostat (Cosier & Glazer, 1986) operating at 100.0 (1) K.
Geometry. All esds (except the esd in the dihedral angle between two l.s. planes) are estimated using the full covariance matrix. The cell esds are taken into account individually in the estimation of esds in distances, angles and torsion angles; correlations between esds in cell parameters are only used when they are defined by crystal symmetry. An approximate (isotropic) treatment of cell esds is used for estimating esds involving l.s. planes.
Refinement. Refinement of F^2^ against ALL reflections. The weighted R-factor wR and goodness of fit S are based on F^2^, conventional R-factors R are based on F, with F set to zero for negative F^2^. The threshold expression of F^2^ > 2sigma(F^2^) is used only for calculating R-factors(gt) etc. and is not relevant to the choice of reflections for refinement. R-factors based on F^2^ are statistically about twice as large as those based on F, and R- factors based on ALL data will be even larger.

### Fractional atomic coordinates and isotropic or equivalent isotropic displacement parameters (Å^2^)

**Table:**

	*x*	*y*	*z*	*U*_iso_*/*U*_eq_	
S1	0.0080 (5)	−0.00421 (15)	0.6539 (3)	0.0264 (5)	
Br1	0.93512 (13)	0.44899 (5)	1.15745 (9)	0.0234 (3)	
N1	−0.0050 (18)	0.2320 (5)	0.6525 (9)	0.0191 (15)	
N2	−0.2131 (19)	0.1740 (6)	0.5631 (6)	0.0203 (16)	
N3	−0.420 (2)	0.0529 (5)	0.4417 (8)	0.0308 (18)	
N4	−0.5398 (18)	0.1444 (6)	0.3863 (7)	0.0288 (16)	
C1	0.406 (2)	0.2109 (6)	0.9745 (7)	0.0188 (15)	
H1A	0.3374	0.1478	0.9953	0.023*	
C2	0.592 (2)	0.2704 (6)	1.0655 (7)	0.0212 (16)	
H2A	0.6494	0.2466	1.1462	0.025*	
C3	0.6906 (19)	0.3639 (6)	1.0365 (7)	0.0193 (16)	
C4	0.622 (2)	0.3988 (6)	0.9129 (7)	0.0215 (16)	
H4A	0.7033	0.4603	0.8919	0.026*	
C5	0.4312 (19)	0.3397 (6)	0.8237 (7)	0.0175 (15)	
H5A	0.3756	0.3639	0.7432	0.021*	
C6	0.319 (2)	0.2450 (7)	0.8506 (8)	0.0194 (18)	
C7	0.106 (2)	0.1867 (6)	0.7562 (8)	0.0178 (18)	
C8	0.006 (2)	0.0807 (7)	0.7828 (9)	0.0220 (18)	
H8A	−0.2194	0.0815	0.8044	0.026*	
H8B	0.1608	0.0559	0.8540	0.026*	
C9	−0.225 (3)	0.0749 (8)	0.5464 (9)	0.026 (2)	
C10	−0.406 (2)	0.2173 (7)	0.4581 (9)	0.0256 (14)	
C11	−0.458 (2)	0.3240 (7)	0.4400 (9)	0.0256 (14)	
H11A	−0.5670	0.3365	0.3564	0.038*	
H11B	−0.2431	0.3574	0.4552	0.038*	
H11C	−0.5981	0.3484	0.4966	0.038*	

### Atomic displacement parameters (Å^2^)

**Table:**

	*U*^11^	*U*^22^	*U*^33^	*U*^12^	*U*^13^	*U*^23^
S1	0.0275 (13)	0.0247 (10)	0.0279 (10)	0.0019 (7)	0.0075 (10)	−0.0060 (9)
Br1	0.0201 (4)	0.0356 (4)	0.0131 (4)	−0.0002 (4)	−0.0007 (2)	−0.0050 (4)
N1	0.013 (4)	0.025 (3)	0.018 (3)	0.002 (2)	−0.001 (3)	−0.005 (3)
N2	0.025 (4)	0.028 (4)	0.008 (3)	0.002 (3)	0.002 (3)	0.002 (3)
N3	0.033 (5)	0.038 (4)	0.022 (4)	−0.006 (3)	0.006 (3)	−0.010 (3)
N4	0.025 (4)	0.047 (5)	0.017 (3)	−0.005 (3)	0.009 (3)	−0.007 (3)
C1	0.024 (4)	0.023 (4)	0.009 (4)	−0.001 (3)	0.003 (3)	0.002 (3)
C2	0.020 (4)	0.032 (4)	0.011 (4)	0.007 (3)	0.001 (3)	−0.003 (3)
C3	0.015 (4)	0.029 (4)	0.014 (4)	0.006 (3)	0.004 (3)	0.001 (3)
C4	0.022 (4)	0.026 (4)	0.016 (4)	0.002 (3)	0.003 (3)	0.004 (3)
C5	0.017 (4)	0.027 (4)	0.008 (3)	0.001 (3)	0.002 (3)	0.001 (3)
C6	0.023 (4)	0.029 (4)	0.007 (4)	0.006 (3)	0.003 (3)	0.000 (3)
C7	0.026 (5)	0.013 (4)	0.017 (5)	0.006 (3)	0.009 (4)	0.001 (3)
C8	0.027 (5)	0.018 (4)	0.022 (5)	−0.003 (3)	0.009 (4)	0.001 (3)
C9	0.025 (5)	0.032 (5)	0.020 (5)	−0.003 (3)	0.005 (4)	−0.006 (3)
C10	0.015 (3)	0.048 (4)	0.016 (3)	−0.004 (3)	0.007 (2)	0.000 (3)
C11	0.015 (3)	0.048 (4)	0.016 (3)	−0.004 (3)	0.007 (2)	0.000 (3)

### Geometric parameters (Å, º)

**Table:**

S1—C9	1.737 (11)	C2—H2A	0.9300
S1—C8	1.813 (10)	C3—C4	1.413 (11)
Br1—C3	1.892 (8)	C4—C5	1.384 (11)
N1—C7	1.298 (13)	C4—H4A	0.9300
N1—N2	1.408 (11)	C5—C6	1.395 (13)
N2—C9	1.342 (14)	C5—H5A	0.9300
N2—C10	1.399 (12)	C6—C7	1.454 (12)
N3—C9	1.307 (13)	C7—C8	1.519 (13)
N3—N4	1.417 (11)	C8—H8A	0.9700
N4—C10	1.311 (12)	C8—H8B	0.9700
C1—C2	1.391 (11)	C10—C11	1.455 (14)
C1—C6	1.417 (11)	C11—H11A	0.9600
C1—H1A	0.9300	C11—H11B	0.9600
C2—C3	1.370 (12)	C11—H11C	0.9600
			
C9—S1—C8	94.0 (4)	C5—C6—C7	120.8 (7)
C7—N1—N2	115.1 (6)	C1—C6—C7	121.7 (8)
C9—N2—C10	107.4 (8)	N1—C7—C6	116.3 (8)
C9—N2—N1	130.2 (8)	N1—C7—C8	122.9 (8)
C10—N2—N1	121.4 (8)	C6—C7—C8	120.7 (8)
C9—N3—N4	106.7 (7)	C7—C8—S1	113.8 (7)
C10—N4—N3	108.5 (7)	C7—C8—H8A	108.8
C2—C1—C6	121.0 (8)	S1—C8—H8A	108.8
C2—C1—H1A	119.5	C7—C8—H8B	108.8
C6—C1—H1A	119.5	S1—C8—H8B	108.8
C3—C2—C1	120.1 (8)	H8A—C8—H8B	107.7
C3—C2—H2A	120.0	N3—C9—N2	110.2 (9)
C1—C2—H2A	120.0	N3—C9—S1	129.0 (8)
C2—C3—C4	120.5 (7)	N2—C9—S1	120.7 (7)
C2—C3—Br1	121.8 (6)	N4—C10—N2	107.1 (9)
C4—C3—Br1	117.7 (6)	N4—C10—C11	128.2 (9)
C5—C4—C3	118.7 (7)	N2—C10—C11	124.6 (8)
C5—C4—H4A	120.6	C10—C11—H11A	109.5
C3—C4—H4A	120.6	C10—C11—H11B	109.5
C4—C5—C6	122.2 (7)	H11A—C11—H11B	109.5
C4—C5—H5A	118.9	C10—C11—H11C	109.5
C6—C5—H5A	118.9	H11A—C11—H11C	109.5
C5—C6—C1	117.4 (8)	H11B—C11—H11C	109.5
			
C7—N1—N2—C9	−26.3 (14)	C1—C6—C7—C8	−8.3 (13)
C7—N1—N2—C10	166.6 (8)	N1—C7—C8—S1	43.3 (11)
C9—N3—N4—C10	1.6 (11)	C6—C7—C8—S1	−140.3 (7)
C6—C1—C2—C3	0.9 (12)	C9—S1—C8—C7	−48.8 (7)
C1—C2—C3—C4	−3.7 (12)	N4—N3—C9—N2	0.5 (11)
C1—C2—C3—Br1	178.6 (6)	N4—N3—C9—S1	−177.5 (7)
C2—C3—C4—C5	4.8 (12)	C10—N2—C9—N3	−2.2 (12)
Br1—C3—C4—C5	−177.3 (6)	N1—N2—C9—N3	−170.6 (9)
C3—C4—C5—C6	−3.3 (12)	C10—N2—C9—S1	176.0 (7)
C4—C5—C6—C1	0.6 (13)	N1—N2—C9—S1	7.5 (14)
C4—C5—C6—C7	177.0 (7)	C8—S1—C9—N3	−154.5 (10)
C2—C1—C6—C5	0.7 (13)	C8—S1—C9—N2	27.7 (9)
C2—C1—C6—C7	−175.7 (8)	N3—N4—C10—N2	−2.8 (10)
N2—N1—C7—C6	−179.6 (7)	N3—N4—C10—C11	−179.4 (9)
N2—N1—C7—C8	−3.0 (13)	C9—N2—C10—N4	3.1 (10)
C5—C6—C7—N1	−7.9 (13)	N1—N2—C10—N4	172.8 (8)
C1—C6—C7—N1	168.4 (9)	C9—N2—C10—C11	179.8 (9)
C5—C6—C7—C8	175.5 (9)	N1—N2—C10—C11	−10.5 (13)

### Hydrogen-bond geometry (Å, º)

**Table:**

*D*—H···*A*	*D*—H	H···*A*	*D*···*A*	*D*—H···*A*
C8—H8*A*···N3^i^	0.97	2.56	3.185 (12)	122
C8—H8*B*···N3^ii^	0.97	2.31	3.191 (12)	151

Symmetry codes: (i) *x*, −*y*, *z*+1/2; (ii) *x*+1, −*y*, *z*+1/2.
